# The Interaction between Anesthetic Isoflurane and Model-Biomembrane Monolayer Using Simultaneous Quartz Crystal Microbalance (QCM) and Quartz Crystal Impedance (QCI) Methods

**DOI:** 10.3390/membranes14030062

**Published:** 2024-02-27

**Authors:** Yasushi Yamamoto, Daiki Ito, Honoka Akatsuka, Hiroki Noguchi, Arisa Matsushita, Hyuga Kinekawa, Hirotaka Nagano, Akihiro Yoshino, Keijiro Taga, Zameer Shervani, Masato Yamamoto

**Affiliations:** 1 Department of Life Science and Applied Chemistry, Graduate School of Engineering, Nagoya Institute of Technology, Nagoya 466-8555, Japan; 2 Food & Energy Security Research & Product Centre, Sendai 980-0871, Japan; 3 Department of Chemistry, School of Arts and Sciences, Showa University, Fujiyoshida 403-0005, Japan

**Keywords:** quartz crystal microbalance (QCM), quartz crystal impedance (QCI), simultaneous measurement, isoflurane, dipalmitoyl phosphatidyl choline (DPPC), palmitic acid (PA), alamethicin (Al), *ß*-Lactoglobulin (*_ß_*LG), monolayer, anesthetic hydrate, physisorption, interfacial viscosity, mechanism of anesthesia

## Abstract

The interaction between anesthetic Isoflurane (Iso) and model-biomembrane on the water surface has been investigated using quartz crystal microbalance (QCM) and quartz crystal impedance (QCI) methods. The model-biomembranes used were dipalmitoyl phosphatidyl choline (DPPC), DPPC-palmitic acid (PA) mixture (DPPC:PA = 8:2), DPPC-Alamethicin (Al) mixture (DPPC:Al = 39:1), and DPPC-*β*-Lactoglobulin (*_β_*LG) mixture (DPPC:*_β_*LG = 139:1) monolayers, respectively. The quartz crystal oscillator (QCO) was attached horizontally to each monolayer, and QCM and QCI measurements were performed simultaneously. It was found that Iso hydrate physisorbed on each monolayer/water interface from QCM and changed those interfacial viscosities from QCI. With an increase in Iso concentration, pure DPPC, DPPC-PA mixed, and DPPC-Al mixed monolayers showed a two-step process of Iso hydrate on both physisorption and viscosity, whereas it was a one-step for the DPPC-*_β_*LG mixed monolayer. The viscosity change in the DPPC-*_β_*LG mixed monolayer with the physisorption of Iso hydrate was much larger than that of other monolayers, in spite of the one-step process. From these results, the action mechanism of anesthetics and their relevance to the expression of anesthesia were discussed, based on the “release of interfacial hydrated water” hypothesis on the membrane/water interface.

## 1. Introduction

Anesthesia is a phenomenon that temporarily reduces nerve function, resulting in the loss of perception and consciousness, including pain. So far, various ideas and trials have been performed with two main axes of analgesic and unconsciousness methods, and now anesthesia is positioned as “invasive control medical science” that controls and manages the body’s protective response against surgical invasion of the body. All surgeries can be performed if the problems of safety are resolved with this anesthesia, but the action mechanism has not yet been elucidated. The theory of anesthesia, which started with the lipoid theory that anesthetics act on the lipid part of the biomembrane [1,2], has now developed into the protein theory that anesthetics act directly on the biomembrane proteins [3,4,5]. The elucidation of the three-dimensional structure of biomembrane protein subunits [6] was supposed to put an end to the anesthetic controversy, but it did not lead to the identification of the action site of anesthetics. On the other hand, in 2020, it was reported that lipid rafts in the biomembrane were destroyed by the action of anesthetics [7], and the current tendency is to return to the lipid theory. It is necessary to elucidate how anesthetics act on the lipids, membrane proteins, and their surrounding area change the biomembrane structure and how the biomembrane function is affected by the action of anesthetics and leads to the expression of anesthesia.

Various model-biomembranes, such as monolayers and bilayers, micelles, vesicles, and liposormes, are used for the elucidation of membrane function [8,9,10,11,12,13,14,15,16] including the anesthesia phenomenon. Among them, the monolayers formed on the water surface have the following characteristics: amphiphilic molecules arrange their hydrophobic moieties toward the air phase and their hydrophilic moieties toward the water subphase. It is possible to form an easy and stable monomolecular flat layer and to control the arbitral number of monolayer molecules. Moreover, they give half the structure of the biomembrane [17]. It is expected that the monolayers provide the fundamental clue to the interaction between biomembrane and anesthetics at the molecular level. Among various interfacial chemistry techniques for investigating physicochemical properties including the mechanism of anesthesia, the quartz crystal oscillator (QCO) method is a powerful and useful tool for such investigations in liquid phases [18,19]. The QCO method is classified into quartz crystal microbalance (QCM) and quartz crystal impedance (QCI), respectively. QCM can detect a mass change in the nanogram-order range according to Sauerbrey’s equation [20], which is used to analyze the adsorption phenomena on the QCO such as oxidation/reduction reaction, metal ion binding to Langmuir monolayer, and molecular recognition of DNA strands and lipids [21,22,23,24,25,26]. QCI can also detect and estimate a change in viscosity and elasticity of adsorbed organic and inorganic layers on the QCO independent of adsorbed mass [27] and be used in the analysis of changes in properties of adsorbed layers on the QCO such as hybridization of DNA [28], phase transition of Langmuir–Blodgett film [29], and protein precipitation denaturation [30,31]. It is expected that the use of QCM and QCI methods would help the elucidation of our targeted anesthesia phenomenon.

In this study, we have investigated the interaction between various pure- and mixed-monolayers including model proteins on the water surface (model-biomembrane) and anesthetic isoflurane using the QCO method (QCM and QCI) as one of the physicochemical methods. We decided to adopt four samples of dipalmitoyl phosphatidyl choline (DPPC), palmitic acid (PA), alamethicin (Al), and *β*-Lactoglobulin (*_β_*LG) as model-biomembrane substances. DPPC and PA are typical amphiphilic molecules that are the main constituents of biomembrane. Many studies, such as membrane morphology and interaction with various functional substances, have been conducted using those molecules as model-biomembrane [32]. Al and *_β_*LG are also typical peptide proteins composed of many amino acid residues. It is known that the Al molecule has a bent-type *α*-helix rigid structure, whereas the *_β_*LG molecule has a globular type consisting mainly of a *β*-sheet structure. Many studies are now being conducted with a focus on those structural specificities and the interaction with lipids from the viewpoint of bio-membrane function [33,34,35]. The QCO for QCM and QCI measurements was attached horizontally to each monolayer, and QCM and QCI measurements were performed simultaneously. Based on the obtained results, we have also discussed the relevance of anesthetic phenomena from the viewpoint of the adsorption of anesthetics to the biomembrane interface and the change in the biomembrane structure. In addition to the lipids listed above, we selected typical model proteins (rigid Alamethicin and soft (swollen) *_β_*LG) as basic materials. Using the QCO method, the above monolayers prepared on the water surface were investigated to extend the current knowledge about the model biological membrane structures.

## 2. Experimental

### 2.1. Materials

**Figure 1:**
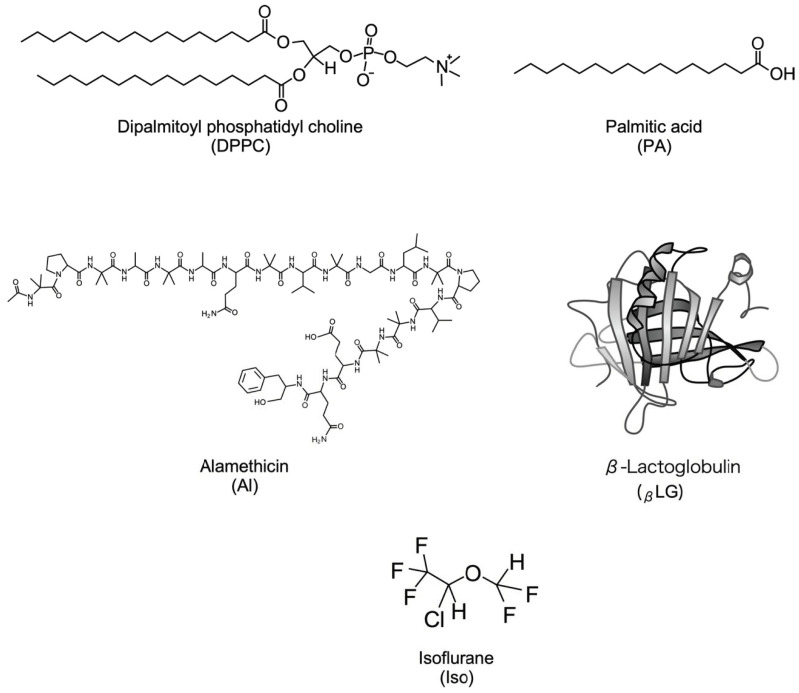
The structure of monolayer components and anesthetic used: DPPC, PA, Al, *_β_*LG [36], and Iso.

Dipalmitoyl phosphatidylcholine (DPPC, purity > 99%) and palmitic acid (PA, purity > 99%) were obtained from Avanti Polar Lipids Inc. (Alabaster, AL, USA) and Sigma-Aldrich Inc. (St. Louis, MO, USA), respectively. Sigma-Aldrich Inc. (St. Louis, MO, USA) provided the β-Lactoglobulin (bovine milk (_β_LG, purity > 90%)), whereas LKT Laboratories Inc. (St Paul, MN, USA) supplied alamethicin (Al, purity 99%). These chemicals were used without further purification. Chloroform with a purity > 99.0% and methanol (above 99.8% pure), the two spreading solvents used for monolayer preparation and volatile anesthetic isoflurane (Iso, grade > 98% pure) were acquired from FUJIFILM Wako Pure Chemical Corporation located in Osaka, Japan. The concentrations of isoflurane solution prepared in water were kept in the range of 1–8 mM. For *_β_*LG, we used ultrapure water refined from Direct-Q UV3 of G = 16.8 MΩ∙cm by Merck Millipore (Belmopán; Belize) as a solvent. Furthermore, it was used as a water subphase for the following monolayer formation. The structures of monolayer substances and Iso is shown in Figure 1 [36].

### 2.2. Monolayer Formation

In this research, various pure- and mixed monolayers were used for the interaction between anesthetic Iso and each monolayer. To make dropping solutions of 0.5 mM of DPPC and PA, the components were dissolved in chloroform for each pure DPPC, PA, and DPPC-PA mixed monolayer preparation. DPPC and Al mixture for DPPC-Al mixed monolayer was dissolved in chloroform–methanol mixed solvent (*v*_chl_:*v*_met_ = 2:1) to prepare a dropping solution of 0.5 mM. *_β_*LG for DPPC-*_β_*LG mixed monolayer was dissolved in ultrapure water to prepare a dropping solution of 50 µM. For each monolayer of pure DPPC, PA, mixed DPPC-PA, and mixed DPPC-Al, each dropping solution was put on the water surface using a 100 μL microsyringe (Ge-0583-04), a product of Hamilton Corporation (Reno, NV, USA), to prepare each monolayer.

In previous articles [16,37], more details of the monolayer preparation were presented. The experimental details of the monolayer formation have been reported previously [16,37]. After gently spreading a 1 μL droplet of the dropping solution on the water surface, the next drop was added after ≥1 min to ensure that the previous droplet components expanded sufficiently on the water surface. The surface tension was monitored until it reached a constant value, indicating that molecular expansion and equilibrium were attained on the water surface, as shown in Section 2.3.1. The emergence of a droplet lens on the surface of the water and a constant surface tension value showed that the monolayer formation was completed. The DPPC:PA = 8:2 and DPPC:Al = 39:1 mole ratios of the DPPC-PA mixed monolayer and DPPC-Al mixed monolayer, respectively, were investigated. For the monolayer of pure *_β_*LG, at which the solvent was water, the monolayer formation was performed as same as the above method: a 100 μL microsyringe was used to drop *_β_*LG/water solution on the water surface at intervals of one minute. After the continuous dropping, the completion of monolayer formation was confirmed when the surface tension value became constant despite the dropping of the solution. For the mixed monolayer of DPPC and *_β_*LG mixture, the monolayer formation was performed according to the work in [38]: at first, the corresponding mixing ratio volume of *_β_*LG/water solution was dropped on the water surface with a 100 μL microsyringe and the time of dropping interval was 1 min. After the continuous dropping and the finish of dropping of *_β_*LG/water solution, the corresponding mixing ratio volume of DPPC/Chloroform solution was subsequently dropped on the water surface, including scattered *_β_*LG molecules, as was the case with the above pure DPPC monolayer. The completion of the DPPC-*_β_*LG mixed monolayer was confirmed by the final drop of the DPPC/Chloroform solution and the appearance of the droplet lens on the water surface. A mole ratio of the DPPC-*_β_*LG mixed monolayer was performed on DPPC:*_β_*LG = 129:1. After each stock solution of the membrane components was added to the water surface, the solvents chloroform or methanol evaporated due to their volatile nature, leaving the lipids and the additives on the water surface to make the monolayer.

### 2.3. Methods

#### 2.3.1. Surface Tension Measurement (STm) and Apparatus

A Surface Tensiometer (model: CBVP-A3, manufactured by Kyowa Interface Science Corporation Limited, Japan) attached to a platinum Wilhelmy was employed to conduct the surface tension measurement (STm) of prepared pure and mixed monolayers [16,37]. As mentioned in Section 2.2, each solution for monolayer formation was spread on the purified water surface, and the surface tension (ST) was recorded after the dropping of each droplet and sufficient expansion. Surface pressure (π) was worked out from the measured ST using the *π* = *γ*_0_ − *γ*, where *γ*_0_ is the value of ST of water and *γ* is the value of ST under the existence of monolayer substance. The spreading volume (molecule numbers) was used to determine the π values of each monolayer, which was subsequently depicted by a π-A isotherm curve. The temperature of the measurement instrument was maintained at 26.0 ± 0.2 °C. The measurement error was within the limit ± 0.2 mN/m and each experiment was repeated more than 3 times. The area per lipid (one molecule) was calculated by dividing the surface area of the used glass petri dish by the number of lipid molecules dropped on the water surface in the dish. Our laboratory computer has a short Excel calculation program to give the number of molecules each drop has, which was used to work out the area per lipid molecule.

#### 2.3.2. Quartz Crystal Oscillator (QCO) Method and Apparatus

Previous articles [16,39,40,41] described the quartz crystal microbalance (QCM) and quartz crystal impedance (QCI) apparatus and measurement method. A short explanation is given below: a quartz crystal oscillator (QCO) with a frequency of 5 MHz and an Au electrode area of 137 mm^2^ was bought from Stanford Research Systems (SRS) Inc. (Sunnyvale, CA, USA). The QCO attached to the O-ring, which corresponded to the diameter of QCO on the bottom of a poly(tetrafluoroethylene) holder including two electrode contacts for QCO, was gently moved (10 µm/s) with the help of a micrometer and connected horizontally to the monolayer that formed on the water surface. The hydrophobic interaction that occurred between the hydrophobic group of each monolayer and the hydrophobic Au electrode surface enables each monolayer to be physically bound to the Au electrode surface of the QCO [42,43,44]. From the tube attached to the trough, anesthetic Iso was injected from the bottom of the poly(tetrafluoroethylene) trough that was part of the apparatus. An ultra-slow magnetic stirrer (HP90740/HP70100, ISIS Co., Ltd., Osaka, Japan) was used to dissolve the added Iso (droplets) into the water subphase by slowly rotating the stirring bar at a rate of 1 r/s. Because of the extremely slow stirring, the mixing of Iso did not affect the stability of the monolayer. Using a homemade double-layered water bath, the temperature was kept at 26.00 ± 0.01 °C during the QCM and QCI experiments.

For the QCM and QCI measurements, the frequency *F* of the QCO (QCM) and the resistance *R* in the QCO circuit (QCI) were measured simultaneously by commercialized QCM200 apparatus (SRS inc., Sunnyvale, CA, USA) and measurements were conducted at 10 s intervals, which were controlled using a personal computer (OPTIPLEX9020; Dell Technologies Japan Inc., Tokyo, Japan). *R* corresponds to the viscosity change at the QCO interface including the attached monolayer, different from commercialized QCM-D which stops QCO resonance instantaneously and observes vibration damping (dissipation: ΔD). We focused on *R* from the viewpoint of membrane fluidity [45,46]. After the stabilization of *F* and *R* contacting the monolayer within ±0.2 Hz for QCM and ±0.02 Ω for QCI longer than 12 h, respectively, anesthetic Iso was injected slowly at the rate of 1 µL/s using a 100 µL microsyringe. The change in *F* and *R* was observed and recorded before and after the addition of Iso, and it was also recorded after more than 5 h to let the system attain equilibrium. QCM and QCI simultaneous measurements were conducted more than two times to maintain the reproducibility of the data. The experimental errors were noticed within ±0.4 Hz for QCM and ±0.04 Ω for QCI.

## 3. Results and Discussion

### 3.1. π-A Isotherm Curve of Each Monolayer

**Figure 2:**
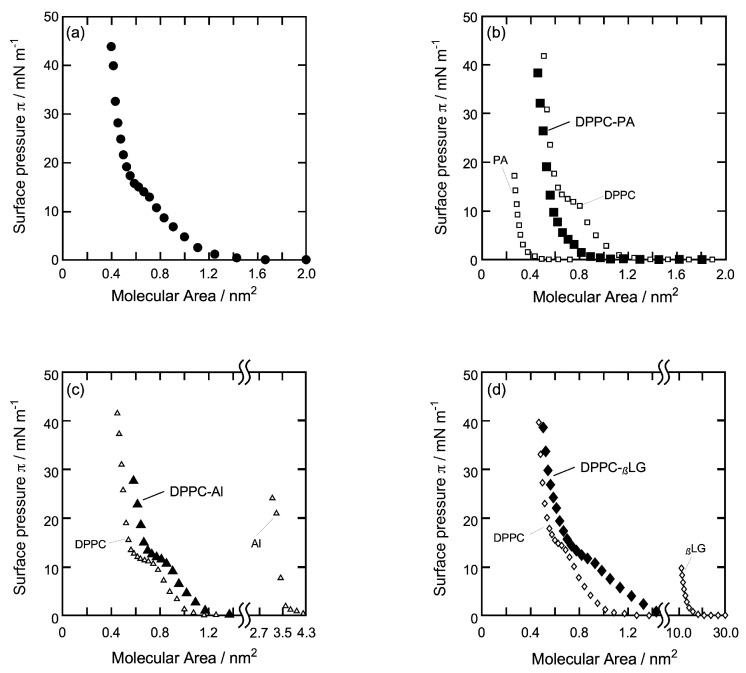
*π*-*A* isotherm curves of each monolayer used in the QCM and QCI measurements. (**a**): DPPC, (**b**): DPPC-PA (8:2), (**c**): DPPC-Al (39:1), and (**d**): DPPC-*_β_*LG (129:1). Monolayers prepared by the dropping method at 26 °C. (**b**–**d**) also involves *π*-*A* isotherm curves of pure monolayer before the mixing of each sample as opened symbol (**b**): DPPC and PA, (**c**): DPPC and Al, (**d**): DPPC and *_β_*LG.

In order to clarify the properties of each monolayer on which Iso molecules act, the monolayer morphology was analyzed by the *π*-*A* isotherm curve obtained from surface tension measurement (STm). Figure 2 shows *π*-*A* isotherm curves of each monolayer (Figure 2a: DPPC, Figure 2b: DPPC-PA (8:2), Figure 2c: DPPC-Al (39:1), Figure 2d: DPPC-*_β_*LG (129:1)) recorded by the dropping method at 26 °C used in the QCM and QCI measurements. The horizontal axis represents the molecular area (*A*) calculated from molecular numbers in the dropping volume, and the vertical axis represents surface pressure (*π*) at the equilibrium state after dropping the sample solution on the water surface. Figure 2b–d also involves *π*-*A* isotherm curves of pure monolayer before the mixing of each sample shown as opened symbols (Figure 2b: DPPC and PA, Figure 2c: DPPC and Al, Figure 2d: DPPC and *_β_*LG) and are normalized based on the *π*-*A* isotherm curve of the DPPC monolayer (Figure 2a). Each monolayer is formed from the lower right to the upper left of each curve.

As shown in Figure 2a, *π* of pure DPPC increased gradually from *ca.* 1.2 nm^2^ molecular area, and after an unclear and plateau range at around *π* = 14 mN/m, increased up to *π* = 44 mN/m. The plateau range corresponds to the two-dimensional phase transition from a liquid-expanded (LE) to a liquid-condensed (LC) state. The limiting molecular area *A*_0_ was 0.59 nm^2^/molecule (*ca.* 40 mN/m) and 23% larger than that obtained by the compression method (0.48 nm^2^/molecule) [32,47]. The present curve was in close agreement with those we have reported previously [37], indicating that the DPPC monolayer is in a semi-expanded state and has a flexible and fluid structure. The dropping method allows the hydrophilic group of DPPC molecules and water molecules to make a structure that maintains the most comfortable hydrogen-bonding network due to the hydrophilic interaction, including the flexibility of two alkyl chains in DPPC molecules [16,37].

In Figure 2b, *π* of the DPPC-PA mixture increased gradually from *ca.* 1.0 nm^2^ molecular area, and after a small flection point at *π* = 8 mN/m, increased up to *π* = 39 mN/m. The flection point corresponds to the LE-LC transition, although there was no plateau range as pure DPPC monolayer. *A*_0_ was 0.58 nm^2^/molecule (*ca.* 35 mN/m) and similar to that of pure DPPC monolayer (0.59 nm^2^/molecule). The value was also larger than that of the ideal mixed state (0.53 nm^2^/molecule) calculated from each *A*_0_ of pure DPPC and PA (0.30 nm^2^/molecule) monolayer. This positive deviation indicates that the DPPC-PA mixed monolayer is in a partially expanded state that has a more flexible and fluid structure than the pure DPPC monolayer. This may be also due to the specific flexibility of the alkyl chain in PA molecules including gauche conformation [48].

As shown in Figure 2c, *π* of the DPPC-Al mixture increased gradually from *ca.* 1.2 nm^2^ molecular area, and after an unclear plateau range at around 14 mN/m, it increased up to *π* = 29 mN/m. Similar to the pure DPPC monolayer, the plateau range corresponds to the phase transition from LE to LC state. *A*_0_ was 0.69 nm^2^/molecule (*ca.* 28 mN/m) and slightly larger than that of the ideal mixed state (0.66 nm^2^/molecule) calculated from each *A*_0_ of the pure DPPC and Al (3.5 nm^2^/ molecule) monolayer. The curve shape was similar to that of the pure DPPC monolayer and seemed to shift to the right side of the pure DPPC curve, indicating that the DPPC-Al mixed monolayer is in a semi-expanded state similar to that of the pure DPPC monolayer and has a flexible and fluid structure, although it includes the Al molecule, which possesses the condensation characteristics [49,50,51]. Moreover, the mole ratio of DPPC:Al = 39:1 corresponds to the *A*_0_ ratio (nm^2^) of DPPC:Al = 23:3.5, making it suitable as a model biomembrane of a lipid–protein mixture. When the antibiotic alamethicin (Al) was added to the monolayer, A_0_ increased to 0.69 nm^2^/molecule from 0.66 nm^2^/molecule (pure DPPC monolayer), with the DPPC-Al isotherm shifting to the right. Similarly, antibiotic alamethicin (AI) altered (perturbed) the phase behavior of nonlamellar lipid phase systems, as reported by Keller et al. [52] using X-ray diffraction and nuclear magnetic resonance (NMR). By affecting the curvature properties of a lipid film, alamethicin significantly changed the cubic phase in phase diagrams.

In Figure 2d, *π* of the DPPC-*_β_*LG mixture increased gradually from *ca.* 1.5 nm^2^ molecular area and, after an unclear and plateau range at around 14 mN/m, increased up to *π* = 39 mN/m. As with the pure DPPC monolayer, the plateau range corresponds to the phase transition from LE to LC state. *A*_0_ was 0.68 nm^2^/molecule (*ca.* 35 mN/m) and slightly smaller than that of the ideal mixed state (0.70 nm^2^/molecule) calculated from each *A*_0_ of pure DPPC and *_β_*LG (14.8 nm^2^/ molecule) monolayer. The curve shape was similar but more gently compared to that of pure DPPC monolayer, indicating that DPPC-*_β_*LG mixed monolayer is in an expanded state and has a more flexible and fluid structure compared to that of the DPPC-Al monolayer (Figure 2c). The specific flexibility of *_β_*LG [53,54,55] may contribute to the formation of an expanded mixed monolayer. Moreover, the mole ratio of DPPC:*_β_*LG = 129:1 corresponds to the *A*_0_ ratio (nm^2^) of DPPC:*_β_*LG = 76:14.8, making it suitable as a model biomembrane of a lipid–protein mixture as same as the DPPC-Al mixture. In Figure 2d, the isotherm of neat DPPC and DPPC-βLG overlapped at high surface pressure is due to the structural difference of *_β_*LG compared to PA and Al (Figure 1). *_β_*LG is less structurally compatible at high surface pressure with DPPC as compared to the other two additives. For that reason, the isotherm is not different at high pressure.

### 3.2. Time Dependence of Frequency (QCM) and Resistance (QCI) on the Action of Iso on the DPPC Monolayer

**Figure 3:**
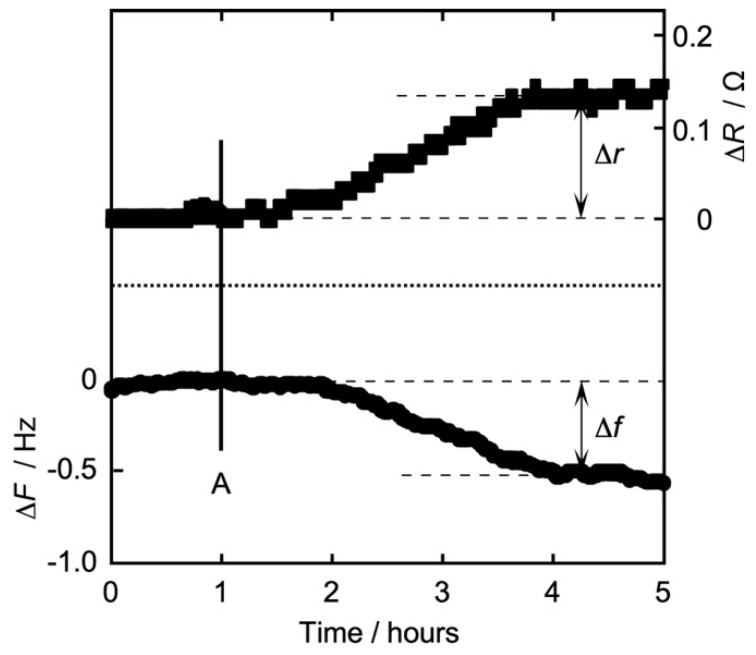
Typical time dependence of simultaneous frequency *F* (QCM) and resistance *R* (QCI) measurements for QCO in contact with a DPPC monolayer after the addition of Iso of 4 mM concentration (bulk subphase) at 26 °C. Lower half side: frequency change Δ*F* (Hz); upper half side: resistance change Δ*R* (Ω), based on each *F* and *R* before the addition of Iso (left and right vertical axes). Line A: time that Isoflurane was added in the water subphase.

Figure 3 shows a typical time dependence of simultaneous frequency Δ*F* (QCM) and resistance Δ*R* (QCI) measurement for QCO in contact with a DPPC monolayer after the addition of Iso of 4 mM concentration (bulk subphase) at 26 °C. Δ*F* and Δ*R* also show the amounts of change when each raw *F* and *R* before the addition of Iso was defined as zero. Figure 3 is also a typical pattern of a change of Δ*F* and Δ*R* observed by the addition of Iso. The lower half side represents the frequency change Δ*F* (Hz, left vertical axis), and the upper half side also represents the resistance change Δ*R* (Ω, right vertical axis). The horizontal axis represents time (h). When a stable baseline was reached for the experiment, Iso was added to the water subphase at the time of line A. After the addition of Iso, Δ*F* decreased gradually due to the response in the physisorption of Iso to the DPPC monolayer/water interface, and Δ*R* also increased gradually due to the change of the interfacial viscosity. Both Δ*F* and Δ*R* approached an equilibrium state after three hours of the addition of Iso. Iso is a typical hydrophobic molecule and possesses a small solubility in water, and the stirring bar is stirred slowly and gently in the water subphase (1 r/s). So, the delayed response of 1 h after the addition probably corresponds to the necessary time for the dissolution and diffusion of Iso molecules into the water subphase and toward the DPPC monolayer/water interface [16,24,25,39,40,41,42,43,44,47,48,56,57,58,59]. The general adsorption phenomenon using QCO is due to a direct chemical bond (chemisorption), and such a chemical reaction occurs faster and in quite a short time [24,25,42,43,56]. A specific part to which Iso is likely to bond is not found in the DPPC molecule (hydrophilic group) at the DPPC monolayer/water interface. So, our research is observing not the chemisorption but the physisorption phenomenon between Iso and each monolayer including DPPC. This means that it takes a long time to reach an equilibrium state. The present time dependence was also very similar to our previous report [59], where Δ*F* and Δ*R* were measured independently using 6 MHz QCO. In the case where the change in each ΔF and ΔR was observed, we decided to define the difference between the values before and after the addition of Iso as Δ*f* for QCM and Δ*r* for QCI, as inserted in Figure 3. In the following section on various monolayers vs. Iso, we have described and discussed the concentration dependence of Iso action on each monolayer using Δ*f* and Δ*r*.

### 3.3. Concentration Dependence of Δf and Δr on the Action of Iso to Each Monolayer

#### 3.3.1. DPPC Monolayer

**Figure 4:**
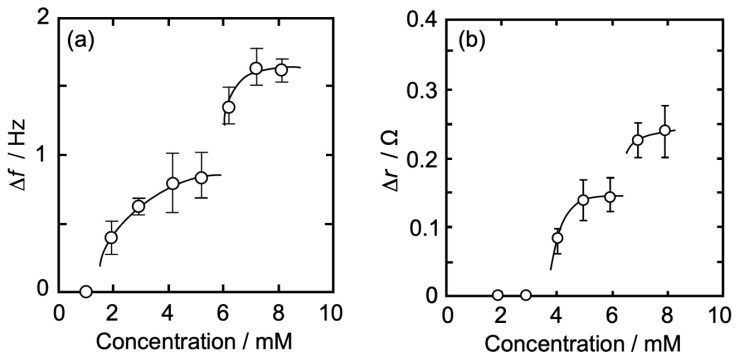
Iso concentration-dependent behavior of Δ*f* and Δ*r* for QCO in contact with a DPPC monolayer (*π* = 35 mN/m) at 26 °C. (**a**): change in Δ*f*, (**b**): change in Δ*r*. Δ*f* of (**a**) also shows the absolute value (positive value) as an amount of change from baseline (Δ*F*) before the addition of Iso.

Figure 4a,b show an Iso concentration-dependent behavior of Δ*f* (QCM, Figure 4a) and Δ*r* (QCI, Figure 4b) for QCO in contact with a DPPC monolayer (*π* = 35 mN/m) at 26 °C. The horizontal axis represents an Iso concentration (bulk subphase), and the vertical axis represents Δ*f* in Figure 4a and Δ*r* in Figure 4b. Δ*f* also shows the absolute value (positive value) as an amount of change from the baseline (Δ*F*) before the addition of Iso.

As can be seen in Figure 4a, there was no change in Δ*f* at an Iso concentration of ≤1 mM, whereas it gradually increased with an increase in concentration at >1 mM and approached asymptotically to a first saturation value of 0.84 Hz at around 5 mM. At >5 mM, Δ*f* started increasing again and approached asymptotically to a second saturation value of 1.61 Hz. The amount of Δ*f* change at each Iso concentration is shown in Table 1. The curve shape of the concentration dependence in Figure 4a was similar to our previous report on the independent QCM measurement using 6 MHz QCO [57], whereas each Δ*f* in this research (Table 1) was a smaller value. Since the Δ*f* change is proportional to the square of the resonance frequency of QCO, it is expected that Δ*f* change in this research would become smaller even if the adsorption amount of Iso molecules was the same between simultaneous and independent measurements.

In Figure 4b, there was no change in Δ*r* at an Iso concentration of ≤3 mM, whereas it gradually increased with the increase in the concentration at >3 mM and approached asymptotically to a first saturation value of 0.14 Ω at around 6 mM. The concentration at which the change in Δ*r* started (4 mM) was higher than that of Δ*f* (2 mM). At >6 mM, Δ*r* started increasing again and approached asymptotically to a second saturation value of 0.25 Ω. The amount of Δ*r* change at each Iso concentration is shown in Table 1. The first curve shape of the concentration dependence and each Δ*r* value (Table 1) in Figure 4b was similar to our previous report on the independent QCI measurement using 6 MHz QCO [58], whereas the second ones were a little different; each Δ*r* value was larger and showed an asymptotic curve. The thickness of 5 MHz QCO is 1.2 times larger than that of 6 MHz QCO. This thickness effect may make possible a stable oscillation of QCO and a sensitive change in resistance in QCO at a concentration of >6 mM.

#### 3.3.2. DPPC-PA Mixed Monolayer

**Figure 5:**
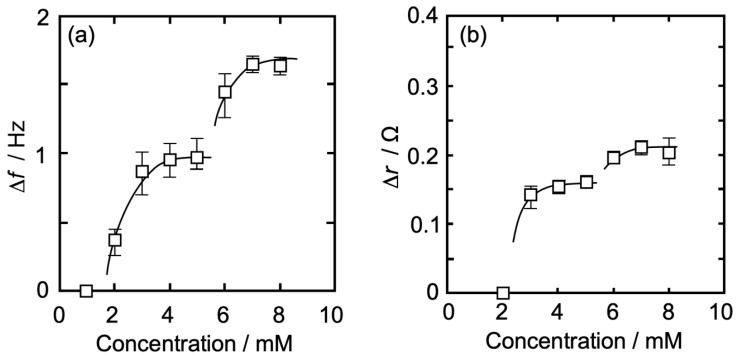
Iso concentration-dependent behavior of Δ*f* and Δ*r* for QCO in contact with a DPPC-PA mixed monolayer (DPPC:PA = 8:2, *π* = 35 mN/m) at 26 °C. The contents of notation are as same as in Figure 4.

Figure 5a,b shows an Iso concentration-dependent behavior of Δ*f* (QCM, Figure 5a) and Δ*r* (QCI, Figure 5b) for QCO in contact with a DPPC-PA mixed monolayer (DPPC:PA = 8:2, *π* = 35 mN/m) at 26 °C. As in Figure 4, the horizontal axis represents an Iso concentration, while the vertical axis represents Δ*f* (absolute value, Figure 5a) and Δ*r* (Figure 5b).

In Figure 5a, there was no change in Δ*f* at an Iso concentration of ≤1 mM, whereas it gradually increased with an increase in concentration at >1 mM and approached asymptotically to a first saturation value of 0.96 Hz at around 5 mM. At >5 mM, Δ*f* started increasing again and approached asymptotically to a second saturation value of 1.75 Hz. The amount of Δ*f* change at each Iso concentration is shown in Table 1. The tendency of Iso concentration dependence was similar to that of pure DPPC monolayer (Figure 4a), but the value change was a little larger at the first curve shape.

In Figure 5b, there was no change in Δ*r* at an Iso concentration of ≤2 mM, whereas it gradually increased with an increase in concentration at >2 mM and approached asymptotically to a first saturation value of 0.15 Ω at around 5 mM. The concentration at which the change in Δ*r* started (3 mM) was higher than that of Δ*f* (2 mM). At >5 mM, Δ*r* started increasing again and approached asymptotically to a second saturation value of 0.20 Ω. The amount of Δ*r* change at each Iso concentration is shown in Table 1. As in Figure 4a, the tendency of Iso concentration dependence was similar to that of pure DPPC monolayer (Figure 4b), but at the first curve shape the change starting concentration was lower (>2 mM) and the change value was larger, at the second curve shape the change starting concentration was lower (>5 mM) and the value change was smaller.

#### 3.3.3. DPPC-Al Mixed Monolayer

**Figure 6:**
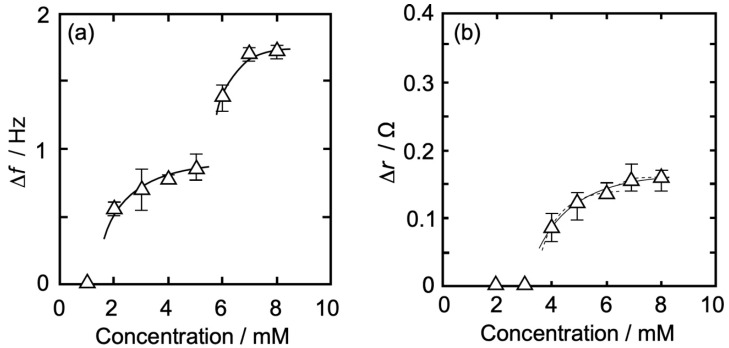
Iso concentration-dependent behavior of Δ*f* and Δ*r* for QCO in contact with a DPPC-Al mixed monolayer (DPPC:Al = 39:1, *π* = 25 mN/m) at 26 °C. The contents of notation are as same as in Figure 4.

Figure 6a,b show an Iso concentration-dependent behavior of Δ*f* (QCM, Figure 6a) and Δ*r* (QCI, Figure 6b) for QCO in contact with a DPPC-Al mixed monolayer (DPPC:Al = 39:1, *π* = 25 mN/m) at 26 °C. In Figure 4 and Figure 5, the horizontal axis represents Iso concentration, while the vertical axis represents Δ*f* (absolute value, Figure 6a) and Δ*r* (Figure 6b). In Figure 6a, there was no change in Δ*f* at an Iso concentration of ≤1 mM, whereas it gradually increased with an increase in concentration at >1 mM and approached asymptotically to a first saturation value of 0.89 Hz at around 5 mM. At >5 mM, Δ*f* started increasing again and approached asymptotically to a second saturation value of 1.74 Hz. The amount of Δ*f* change at each Iso concentration is shown in Table 1. The tendency of Iso concentration dependence was similar to that of the pure DPPC monolayer (Figure 4a).

In Figure 6b, there was no change in Δ*r* at Iso concentration of ≤3 mM, whereas it gradually increased with an increase in concentration at >3 mM. Two patterns are possible: one is a saturation curve that approaches asymptotically to a value of 0.17 Ω until 8 mM (solid line), and the other is two saturation curves that approach asymptotically to a first saturation value of 0.14 Ω at around 6 mM and subsequent a second saturation value of 0.17 Ω at 8 mM (dotted line). In any case, the concentration at which the change in Δ*r* started (>3 mM) was higher than that of Δ*f* (>1 mM). The amount of Δ*r* change at each Iso concentration is shown in Table 1.

#### 3.3.4. DPPC-*_β_*LG Mixed Monolayer

**Figure 7:**
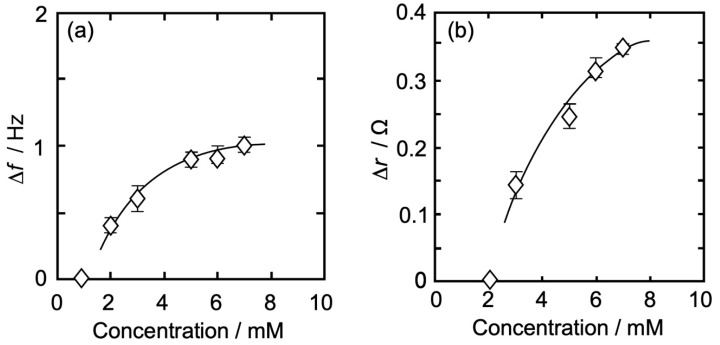
Iso concentration-dependent behavior of Δ*f* and Δ*r* for QCO in contact with a DPPC-*_β_*LG mixed monolayer (DPPC:*_β_*LG = 129:1, *π* = 35 mN/m) at 26 °C. The contents of notation are as same as in Figure 4.

Figure 7a,b show the Iso concentration-dependent behavior of Δ*f* (QCM, Figure 7a) and Δ*r* (QCI, Figure 7b) for QCO in contact with a DPPC-*_β_*LG mixed monolayer (DPPC:*_β_*LG = 129:1, *π* = 35 mN/m) at 26 °C. As in Figure 4, Figure 5 and Figure 6, the horizontal axis represents an Iso concentration, and the vertical axis represents Δ*f* (absolute value, Figure 7a) and Δ*r* (Figure 7b). As can be seen in Figure 7a, there was no change in Δ*f* at an Iso concentration of ≤1 mM, whereas it increased with an increase in concentration at >1 mM and approached asymptotically to a saturation value of 1.00 Hz at around 7 mM. Different from Figure 4a, Figure 5a and Figure 6a, the change in Δ*f* was only one step. The amount of Δ*f* change at each Iso concentration is shown in Table 1.

In Figure 7b, there was no change in Δ*r* at an Iso concentration of ≤2 mM, whereas it increased with an increase in concentration at >2 mM and approached asymptotically to a saturation value of 0.35 Ω at around 7 mM. Different from Figure 4b, Figure 5b and Figure 6b, the change in Δ*r* was only one step, and the amount of value was also nearly twice as large. The amount of Δ*r* change at each Iso concentration is shown in Table 1.

### 3.4. Interaction between Isoflurane and Each Monolayer

Isoflurane (Iso) in this research is a typical inhalation anesthetic and semi-hydrophobic molecule including an oxygen atom (CHF_2_-O-CHCl-CF_3_), and its solubility in water is *ca.* 10 mM at 26 °C [60,61]. In an aqueous solution, water molecules surround one or several Iso molecules and they form isoflurane hydrate aggregates [60,61,62]. It has been reported that enflurane, which is a structural isomer of Iso, is surrounded by 30 water molecules per 1 molecule in the solution [63], so Iso is also considered to contain a similar amount of water molecules. About the action of anesthetics on the model membrane, Yokono et al. have reported using ^1^H-NMR spectroscopy that various anesthetics interact more strongly with the membrane/water interface than the membrane lipid core [64]. By employing 1H- and 19F-NMR spectroscopy, Yoshida and Yoshino’s group also found that the action of anesthetic hydrates changes the structure of the membrane/water interface and releases the interfacial structured water formed on the interface without penetrating into the membrane lipid core [64,65,66]. Based on the above characteristic of anesthetic Iso hydrate and the interfacial action of anesthetics, we discuss the interaction between Iso and each monolayer as follows.

#### 3.4.1. DPPC Monolayer

The present concentration dependence data reported by both the QCM and QCI measurements (Figure 4) were similar to those previously obtained by independent QCM and QCI measurements [16,57]. Therefore, the interaction between Iso and DPPC monolayer is possible to interpret as follows: as shown in Figure 4, on the first saturation range from 1 to 5 mM, Iso hydrates physisorbed on the interfacial structured water formed on the DPPC monolayer/water interface, and the physisorption leads to an increase in Δ*f* as the mass increases. A small amount of Iso hydrates (≤3 mM) does not affect the structured water, thereby there is no change in Δ*r* as the viscosity of the DPPC monolayer/water interface. At >3 mM, the physisorption of Iso hydrates begins to affect the structured water. The interaction between Iso hydrates and the structured water causes “distortion” and “release” of the structured water, and the reconstruction of the structured water, including Iso hydrates, occurs at the interface [63]. This process promotes the interaction between the released hydrophilic groups of DPPC molecules and reduces the distance between those groups. The interfacial reconstruction, including Iso hydrate, also strengthens the interfacial structure and leads to an increase in interfacial viscosity at >3 mM. Since it is possible that hydrophobic alkyl chains in DPPC molecules are attracted to each other by an increase in degrees of freedom of DPPC molecules due to less structured water at the interface resulting in the addition of Iso, consequently the monolayer fluidity and flexibility at the interface will be suppressed. This also leads to an increase in the interfacial viscosity [16,57]. As given in the above section, Δ*r* represents the interfacial viscosity including the structured water. At >3 mM Iso (isoflurane) concentration, physisorption of Iso hydrates alters the structured water, i.e., release and re-arrangement of structured water occurs at the interface. In the presence of Iso, the polar head group of DPPC lipid and Iso molecules (making Iso hydrates) compete or share structured water. In the above section, we mentioned the increase in the interfacial viscosity. However, the water molecules from the lipid polar group are released to make Iso hydrate but the net mass at the interface will increase leading to an increase in the frequency as reported in the Δ*f* plot.

We roughly determine the mean molecular area of Iso hydrate using the value of Δ*f* (0.84 Hz = 14.9 ng/cm^2^ (Sauerbrey’s equation)) from the first saturation values of both Δ*f* (0.84 Hz) and Δ*r* (0.14 Ω) at around 5 mM (Figure 4), as reported by QCM measurement [39,40,57,67]. The effect of the viscosity change (Δ*r*) on the value of Δ*f* was neglected. Yoshino et al. [63] have reported that the amount of hydrated water surrounding one enflurane molecule was estimated to be 30. Iso corresponds to a structural isomer of enflurane, thus we also assumed 30 hydrated water molecules for Iso. The molecular area of the Iso hydrate was calculated as 8.1 nm^2^/one-hydrate at Δ*f* = 0.84 Hz, a little larger than that of preciously reported data (6.2 nm^2^/one-hydrate) [57]. This value corresponds to *A*_0_ of 13.7 DPPC molecules. When Iso hydrate assumes an “elliptical shape”, where the molecular axis of the hydrate is calculated as the major one, the apparent molecular areas along the major and the minor axes are 1.5 nm^2^/one-hydrate and 1.3 nm^2^/one-hydrate, respectively [68]. From these sizes of Iso hydrate, a physisorbed layer of Iso hydrates is certainly formed on the interface, but Iso molecules are not in a dense state and maintain a semi-saturation state.

In the second stage of high concentration at >5 mM (Figure 4), Iso hydrates further physisorb on the interface occupied by somewhat physisorbed Iso hydrates at semi-saturation levels. Two physisorption processes, multi-physisorption of Iso hydrates and promotion of aggregation between hydrates, would occur at the second stage [39,40,41,57,69]. Further physisorption of Iso hydrates corresponding to a greater increase in Δ*f* would result in the Iso hydrate multilayer. The multilayering by Iso hydrates promotes the reconstruction of the interface, including the DPPC monolayer, and consequently leads to an increase in the interfacial viscosity at ≥7 mM. The interface where Iso hydrates are physisorbed is in a locally high concentration state. Iso has the characteristic of forming dimerized or multimerized hydration clusters in the water solution [62]. Those hydrated Iso clusters interact more strongly with the interface, including the DPPC monolayer, resulting in an increase in the interfacial viscosity at ≥7 mM. These two processes are synergistic and may cause a rapid second saturation state compared to the first one.

#### 3.4.2. DPPC-PA Mixed Monolayer

In the first saturation range from 1 to 5 mM (Figure 5), Iso hydrates physisorb on the interfacial structured water formed on the DPPC-PA mixed monolayer/water interface, and the physisorption leads to an increase in Δ*f* as mass increases, as similar to the case of pure DPPC monolayer (Figure 4). As physisorbed Iso hydrates do not affect structured water at concentrations ≤ 2 mM, thereby no change in Δ*r* (representing viscosity) of the mixed monolayer/water interface was observed. At >2 mM, the physisorption of Iso hydrates causes “distortion” and “release” of the structured water formed on the mixed monolayer/water interface, thus causing the interfacial reconstruction and an increase in the interfacial viscosity, including the attraction of alkyl chains in DPPC molecules. The amount of Iso hydrate physisorption at ≥3 mM was larger than that of the pure DPPC monolayer. We have previously reported that the amount of Iso hydrate in the expandable dimyristoyl phosphatidyl choline (DMPC) monolayer was larger than that of the DPPC monolayer and considered that the flexibility of the hydrophilic interface composed of both the hydrophilic group of the DMPC molecules and water molecules leads to an increase in the physisorption amount of Iso hydrates compared to the DPPC monolayer, where the interface was somewhat structured [16,57]. Based on this interpretation, the partially expandable DPPC-PA mixed monolayer, due to the specificity of the PA molecule, would allow more physisorption of Iso hydrate compared to the pure DPPC monolayer. This also leads to an increase in interfacial viscosity at lower concentrations (>2 mM). On the interfacial reconstruction by the physisorption of Iso hydrates, the alkyl chain in PA molecule, including the gauche conformation, may also change into the trans conformation due to the promotion of interaction between alkyl chains in DPPC and PA molecules. The molecular structures of PA and DPPC are similar (PA specificity) to those of other additives used to prepare the monolayers. PA structure most closely resembles the main lipid, DPPC. The concept is used to interpret the data. Actually, we have reported that the DPPC molecule has the property of changing the flexible gauche conformation of the alkyl chain in the DMPC molecule into trans at *x_DMPC_* < 0.3 [70]. Those structural transitions may lead to a larger change in the interfacial viscosity and condensation in the mixed monolayer, resulting in a larger increase in Δ*r* than that of the pure DPPC monolayer.

From the first saturation values of both Δ*f* (0.96 Hz) and Δ*r* (0.15 Ω) at around 5 mM, as shown in Figure 5, we roughly estimated the mean molecular area of Iso hydrate from the value of Δ*f* (0.96 Hz = 17.0 ng/cm^2^), as is the case with the pure DPPC monolayer. The molecular area of the Iso hydrate was calculated as 7.1 nm^2^/one-hydrate. This value corresponds to *A*_0_ of 9.8 DPPC and 2.4 PA molecules. Iso molecules are not in a dense state and maintain a semi-saturation state, although the physisorbed Iso density is a little larger than that of the pure DPPC monolayer. At a high concentration of >5 mM (Figure 5), in the second stage, Iso hydrates further physisorbs on the mixed monolayer/water interface, which is occupied by somewhat physisorbed Iso hydrates at semi-saturation levels, as is the case with the pure DPPC monolayer. Therefore, two processes are expected: multi-physisorption of Iso hydrates and promotion of aggregation between hydrates. Compared to the pure DPPC monolayer, the amount of physisorbed Iso hydrate was almost the same, whereas the change in viscosity was smaller. In the first stage, the increase in viscosity and the larger viscosity change reported at low Iso concentration compared to the pure DPPC monolayer would indicate that the condensation of the mixed monolayer occurs more rapidly than that of the pure DPPC monolayer because the components of the mixed monolayer interact and change their conformation more strongly. Thus, two alkyl chains in the DPPC molecule attract one alkyl chain in the PA molecule more strongly than those in the same DPPC molecule. Due to large condensation occurring in the first stage, there would not be much space left for condensation in the mixed monolayer than in the DPPC monolayer in the second stage. This corresponds to a smaller increase in interfacial viscosity for the mixed monolayer than for the pure DPPC monolayer, even though the amount of phsisorbed Iso hydrates is almost the same between the mixed and pure DPPC monolayers.

#### 3.4.3. DPPC-Al Mixed Monolayer

The interaction between Iso and DPPC-Al mixed monolayers from 1 to 5 mM (Figure 6) was almost the same as the case of pure DPPC monolayers (Figure 4), although Al molecules were included in the mixed monolayer. Al is a rigid molecule, and the mixed monolayer was an ideal mixed state with little interaction with the DPPC molecule (Figure 2c). The amount of Al molecule was also considerably less than that of DPPC molecule (DPPC:Al = 39:1). From these results, the viscosity change of the mixed monolayer in this concentration range is possible to be attributed to DPPC molecules as they are the same as pure DPPC monolayer, therefore Iso hydrates physisorb on the interfacial structured water formed on the mixed monolayer/water interface, and the physisorption leads to an increase in Δ*f* as mass increases. At ≤3 mM, physisorbed Iso hydrates do not affect the structured water, thereby no change in Δ*r* or the viscosity of the mixed monolayer/water interface. At >3 mM, the physisorption of Iso hydrates causes “distortion” and “release” of the structured water of the DPPC/water interface in the mixed monolayer, thereby causing the interfacial reconstruction and an increase in its interfacial viscosity, including the attraction of alkyl chains in DPPC molecules.

From the first saturation values of Δ*f* (0.87 Hz) and corresponding Δ*r* (0.14 Ω) at around 5 mM (Figure 6), we roughly estimated the mean molecular area of Iso hydrate from the value of Δ*f* (0.89 Hz = 15.8 ng/cm^2^), as is the case with the pure DPPC monolayer. The molecular area of the Iso hydrate was calculated to be 7.8 nm^2^/one-hydrate. This value corresponds to the A_0_ of 11 DPPC and 0.3 Al molecules. Iso molecules are not in a dense state and maintain a semi-saturation state, which is the same as that of the pure DPPC monolayer. On high concentrations at ≥6 mM (Figure 6), Iso hydrates further physisorbs on the mixed monolayer/water interface occupied by somewhat physisorbed Iso hydrates at semi-saturation levels, thereby further increasing the Δ*f*. As with the case of pure DPPC and DPPC-PA monolayers, two processes, multi-physisorption of Iso hydrates and promotion of aggregation between hydrates, are expected at this concentration range. About the change in Δ*r*, there are two possibilities, when considered as one saturation curve pattern from low concentration (solid line in Figure 5b), the existence of rigid Al molecules in the DPPC-Al monolayer led to the reconstruction of the DPPC/water interface structure by the physisorption of Iso hydrates until around 6 mM to an interfacial structure similar to that of the pure Al/water interface. This means that there is no more space for further condensation, thereby no more increase in viscosity, even though the above two processes occurred by further physisorption of Iso hydrates at ≥6 mM. When considered as the next (second) saturation curve pattern (dotted line at ≥6 mM in Figure 6b), the above two processes promote the further reconstruction of the DPPC/water interface and thereby an increase in the interfacial viscosity. However, the partial and rigid interfacial structure of the Al molecule interferes with the reconstruction of the DPPC/water interface by further physisorption of Iso hydrates. As a result, the amount of change in viscosity did not reach that of the pure DPPC monolayer. In either possibility, the interface formed by rigid Al molecules would have affected the reconstruction of the DPPC/water interface in the mixed monolayer at ≥6 mM.

#### 3.4.4. DPPC-*_β_*LG Mixed Monolayer

The interaction between Iso and DPPC-*_β_*LG mixed monolayers (Figure 7) was quite different from previous pure DPPC, DPPC-PA, and DPPC-Al monolayers (Figure 4, Figure 5 and Figure 6). Both Δ*f* and Δ*r* showed only one saturation curve on the measured Iso concentration range (1~7 mM). *_β_*LG is a flexible and spherical molecule, and the mixed monolayer was in a flexible and fluid state with little interaction with the DPPC molecule (Figure 2d). The amount of *_β_*LG molecule was also considerably less than that of the DPPC molecule (DPPC:*_β_*LG = 129:1). It has been reported that zwitterionic molecules like DPPC have no ability to induce conformational changes in *_β_*LG molecules, whereas ionic molecules reduce the force of hydrogen bonding between *_β_*LG and water molecule hydrated on the *_β_*LG and promote the intramolecular hydrogen bonding in *_β_*LG and the self-formation from *β*-sheets to *α*-helices [71,72,73]. Since anesthetic molecules, including Iso, have the ability to release hydrated water in the monolayer/water interface by physisorption to the interface [16,57,63,64,65,66,67,69], there are the following possibilities about the change in Δ*r* of the mixed monolayer, including the structural change in *_β_*LG. The physisorption of Iso hydrates occurred at the interfacial structured water that formed on the mixed monolayer/water interface leading to an increase in Δ*f* as mass increases. As Iso hydrates physisorption does not affect the structured water at ≤1 mM, the Δ*r* representing the viscosity of the mixed monolayer/water interface remains unchanged. The physisorption of iso hydrates at >1 mM, results in “distortion” and “release” of the structured water of the mixed monolayer/water interface leading to the reconstruction of the interface and thus increasing the interfacial viscosity. This tendency at the DPPC-*_β_*LG mixed monolayer was similar to that of the DPPC-PA mixed monolayer, but different from that of the pure DPPC and DPPC-Al monolayers. The amount of physisorbed Iso hydrate, on the other hand, was smaller than that of the DPPC-PA mixed monolayer, almost the same as that of the pure DPPC and DPPC-Al monolayers. From those experimental results, it is suggested that the viscosity change at >1 mM on the DPPC-*_β_*LG monolayer can be attributed mainly to the structural change in the *_β_*LG molecule. Therefore, an increase in physisorbed Iso hydrates to the interface with an increase in Iso concentration promotes “distortion” and “release” of the structured water of the mixed monolayer/water interface, and *_β_*LG molecule released from hydrated water molecule changes its structure from *β*-sheets to *α*-helices. There is certainly an attraction of alkyl chains in DPPC molecules to the viscosity change, but the structural change in *_β_*LG molecule contributes largely to the change. In other words, Iso hydrates may physisorb more on the *_β_*LG/water interface than on the DPPC/water interface in the mixed monolayer. This corresponds to a larger increase in Δ*r* than that of the other monolayers. In addition, we approximately calculated the mean molecular area of iso hydrate from the value of Δ*f* (1.10 Hz = 19.5 ng/cm^2^) using the first saturation values of Δ*f* (1.10 Hz) and Δ*r* (0.51 Ω) at about 8 mM (Figure 7), similar to the pure DPPC monolayer. The iso hydrate’s molecular area was determined to be 6.2 nm^2^ per one-hydrate. This number is equivalent to A_0_ for 0.1 *_β_*LG and 9.0 DPPC molecules. Iso molecules are not in a dense state and maintain a semi-saturation state as reported in other monolayers. However, Iso hydrates physisorb relatively faster and more on the *_β_*LG/water interface, and as a result, the average semi-saturation state across the interface may become more concentrated than the other monolayers.

## 4. Conclusions

In the present study, the interaction between anesthetic isoflurane (Iso) and various monolayers (pure DPPC, DPPC-palmitic acid (PA) mixture (DPPC:PA = 8:2), DPPC-Alamethicin (Al) mixture (DPPC:Al = 39:1), and DPPC-*β*-Lactoglobulin (*_β_*LG) mixture (DPPC: *_β_*LG = 139:1)) on the water surface has been investigated using a horizontally QCO-attached device with QCM and QCI simultaneous measurement. All of the monolayers used were in elastic states, as shown by the recorded surface tension measurements (*π*-*A* isotherm curves). It was found that Iso hydrate physisorbed on each monolayer/water interface and changed the interface viscosities. In other words, the morphology of monolayers changed from an expanded to a condensed state. Pure DPPC, DPPC-PA mixed, and DPPC-Al mixed monolayers showed a two-step physisorption process of Iso hydrate around 5–6 mM of Iso concentration, whereas a one-step process of DPPC-*_β_*LG mixed monolayers. The amount of physisorbed Iso hydrates at the first step was interestingly almost the same for all monolayers. Aggregation of Iso hydrate at the interface is suggested at the second physisorption process of pure DPPC, DPPC-PA mixed, and DPPC-Al mixed monolayers. The degree of viscosity change was different depending on the state of each monolayer, particularly the change in the DPPC-*_β_*LG mixed monolayer which was a one-step process and much larger than that of other monolayers.

As mentioned in Section 1, many studies on the anesthesia phenomenon are still being investigated from the viewpoint of interaction between anesthetics and membrane lipids or proteins beyond the frameworks of non-specific and specific theories [3,4,5,7,16,17,39,40,41,57,63,64,65,66,67,69,74,75]. Our study showed that anesthetics, including Iso, have the possibility of acting on both lipid/water and protein/water interfaces in the membrane and changing its properties. According to the “release of interfacial hydrated water” hypothesis [64,76,77,78,79], we suggest the following model of the mechanism of anesthesia: anesthetic hydrates dosed in the body physisorb on the biomembrane/body-fluid interface of nerve cells, at which the biomembrane has a fluid-rich state and a rough interfacial structure. An increase in anesthetic concentration, i.e., an increase in the amount of physisorbed anesthetic hydrates, causes the distortion and release of hydrated water at the interface and promotes the reconstruction of the interfacial structure, including anesthetic molecules, thereby increasing the interfacial viscosity. This reconstruction may trigger the self-structural change of the membrane protein. A semi-saturated network-like hydration structure with anesthetics at the interface also suppresses the interfacial dynamics of the membrane, resulting in reduced interfacial functionality of the membrane. We believe that the source of the mechanism of anesthesia is the reconstruction of lipid/water and protein/water interfaces by the action of anesthetic hydrates on the interface and the accompanying protein self-structural changes. A further increase in anesthetic concentration promotes the aggregation of anesthetic hydrates with an increase in the interfacial viscosity, and the interface is covered by the multilayer of anesthetic hydrates. This leads to the failure of membrane function and cell death. In this study, the interaction between Iso hydrates and DPPC-*_β_*LG mixed monolayer showed that the *_β_*LG structural change occurred. Further detailed investigation using spectroscopic methods such as infrared (IR) spectroscopy would provide deeper insight at the molecular level through the analysis of structural changes in lipids and proteins by the action of anesthetic, thereby understanding the anesthesia phenomenon and the approach to the essence of anesthesia.

## Figures and Tables

**Table 1:** Number of changes in Δ*f* and Δ*r* at each measured Iso concentration. Δ*f*: absolute value (positive value) as an amount of change.

Isoflurane Concentration		2	3	4	5	6	7	8
(mM)								
DPPC	|Δ*f*| (Hz)	0.43	0.63	0.78	0.84	1.38	1.63	1.61
	Δ*r* (Ω)	0.00	0.00	0.10	0.14	0.15	0.23	0.25
DPPC-PA	|Δ*f*| (Hz)	0.36	0.85	0.94	0.96	1.45	1.75	1.75
	Δ*r* (Ω)	0.00	0.14	0.15	0.16	0.20	0.21	0.20
DPPC-Al	|Δ*f*| (Hz)	0.62	0.70	0.79	0.89	1.41	1.73	1.74
	Δ*r* (Ω)	0.00	0.00	0.09	0.13	0.15	0.16	0.17
DPPC-βLG	|Δ*f*| (Hz)	0.41	0.60	-	0.90	0.90	1.00	-
	Δ*r* (Ω)	0.00	0.14	-	0.24	0.30	0.35	-

## Data Availability

Data is contained within the article.
